# 2-Chloro-*N*-(2,3-dimethyl­phen­yl)benzamide

**DOI:** 10.1107/S1600536810024943

**Published:** 2010-07-03

**Authors:** B. Thimme Gowda, Miroslav Tokarčík, Vinola Z. Rodrigues, Jozef Kožíšek, Hartmut Fuess

**Affiliations:** a Department of Chemistry, Mangalore University, Mangalagangotri 574 199, Mangalore, India; b Faculty of Chemical and Food Technology, Slovak Technical University, Radlinského 9, SK-812 37 Bratislava, Slovak Republic; c Institute of Materials Science, Darmstadt University of Technology, Petersenstrasse 23, D-64287 Darmstadt, Germany

## Abstract

In the title compound, C_15_H_14_ClNO, the N—H and C=O bonds in the amide group are *anti* to each other. The amide group is inclined at 60.3 (1)° to the chloro-substituted benzoyl ring and at 59.2 (1)° to the dimethyl-substituted aniline ring. The mean planes through the two benzene rings make a dihedral angle of 7.7 (1)°. In the crystal structure, mol­ecules are linked by inter­molecular N—H⋯O hydrogen bonds, forming chains along [010].

## Related literature

For the preparation of the title compound, see: Gowda, Jyothi *et al.* (2003 ▶). For related structures, see: Gowda, Foro *et al.* (2008 ▶, 2009 ▶); Gowda, Jyothi *et al.* (2003 ▶); Gowda, Tokarčík *et al.* (2009 ▶). For a review of halogen bonding, see: Fourmigué (2009 ▶).
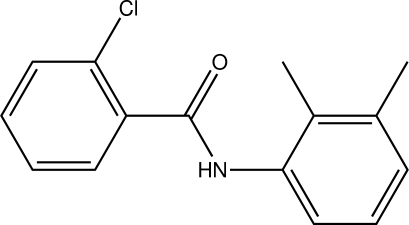


## Experimental

### 

#### Crystal data

C_15_H_14_ClNO
*M* *_r_* = 259.72
Monoclinic, 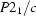

*a* = 13.0108 (5) Å
*b* = 4.9970 (1) Å
*c* = 22.6241 (9) Å
β = 118.553 (4)°
*V* = 1292.01 (9) Å^3^
*Z* = 4
Mo *K*α radiation
μ = 0.28 mm^−1^
*T* = 295 K
0.54 × 0.43 × 0.05 mm

#### Data collection

Oxford Diffraction Gemini R CCD diffractometer
Absorption correction: analytical (*CrysAlis PRO*; Oxford Diffraction, 2009 ▶) *T*_min_ = 0.861, *T*_max_ = 0.985
20701 measured reflections
2293 independent reflections
1959 reflections with *I* > 2σ(*I*)
*R*_int_ = 0.027

#### Refinement

*R*[*F* ^2^ > 2σ(*F* ^2^)] = 0.039
*wR*(*F* ^2^) = 0.102
*S* = 1.06
2293 reflections
166 parameters
H-atom parameters constrained
Δρ_max_ = 0.20 e Å^−3^
Δρ_min_ = −0.20 e Å^−3^

### 

Data collection: *CrysAlis PRO* (Oxford Diffraction, 2009 ▶); cell refinement: *CrysAlis PRO*; data reduction: *CrysAlis PRO*; program(s) used to solve structure: *SHELXS97* (Sheldrick, 2008 ▶); program(s) used to refine structure: *SHELXL97* (Sheldrick, 2008 ▶); molecular graphics: *ORTEP-3* (Farrugia, 1997 ▶) and *DIAMOND* (Brandenburg, 2002 ▶); software used to prepare material for publication: *SHELXL97*, *PLATON* (Spek, 2009 ▶) and *WinGX* (Farrugia, 1999 ▶).

## Supplementary Material

Crystal structure: contains datablocks I, global. DOI: 10.1107/S1600536810024943/tk2683sup1.cif

Structure factors: contains datablocks I. DOI: 10.1107/S1600536810024943/tk2683Isup2.hkl

Additional supplementary materials:  crystallographic information; 3D view; checkCIF report

## Figures and Tables

**Table 1:** Hydrogen-bond geometry (Å, °)

*D*—H⋯*A*	*D*—H	H⋯*A*	*D*⋯*A*	*D*—H⋯*A*
N1—H1*N*⋯O1^i^	0.86	2.23	2.9388 (19)	140

Symmetry code: (i) 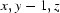.

**Fig. 1.**
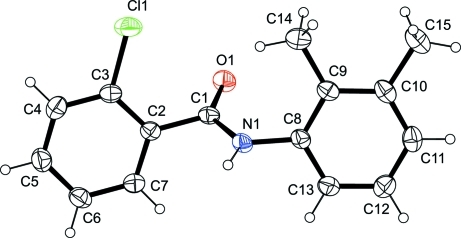
Molecular structure of (I) showing the atom labelling scheme. Displacement ellipsoids are drawn at the 30% probability level and H atoms are represented as small spheres of arbitrary radii.

**Fig. 2.**
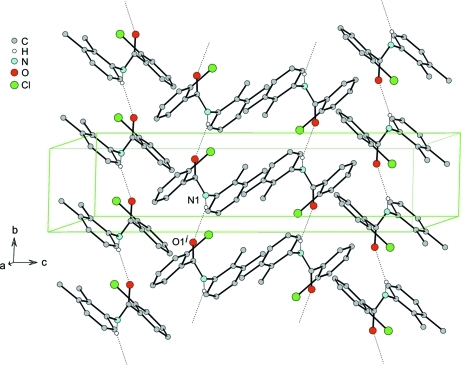
View of the crystal packing of (I), showing the chains of molecules linked by intermolecular N–H···O hydrogen bonds (dashed lines). H atoms not involved in hydrogen bonding have been omitted. Symmetry code (i): x, y - 1, z.

## supplementary crystallographic information

### Comment

To explore the effect of substituents on the structures of benzanilides (Gowda, Foro *et al.*, 2008, 2009; Gowda, Jyothi *et al.*, 2003; Gowda, Tokarčík *et al.*, 2009), in the present work, the structure of 2-chloro-*N*-(2,3-dimethylphenyl)-benzamide (I) has been determined The N—H and C═O bonds in the amide group are *anti* to each other (Fig.1), similar to that observed in 2-chloro-*N*-(phenyl)-benzamide (II) (Gowda, Jyothi *et al.*, 2003), *N*-(2,3-dimethylphenyl)- benzamide (III) (Gowda, Tokarčík *et al.*, 2009), 2-chloro-*N*-(2,3-dichlorophenyl)-benzamide (IV) (Gowda, Foro *et al.*, 2008), and 2-chloro-*N*-(3,5-dimethylphenyl)- benzamide (V) (Gowda, Foro *et al.*, 2009).

The molecular structure of (I) includes a short intramolecular Cl1···O1 contact of 3.1837 (16) Å, which can be interpreted within the concept of halogen bonding (Fourmigué, 2009). The central amide group –NHCO– is inclined at 60.3 (1) ° to the benzoyl ring (C2–C7) and at 59.2 (1) ° to the anilino ring (C8–C13). The mean planes through the two benzene rings make a dihedral angle of 7.7 (1) °. The crystal packing (Fig. 2) is dominated by intermolecular N–H···O hydrogen bonds (Table 1) which link the molecules into the chains extending along the *b *axis.

### Experimental

The title compound was prepared according to the literature method (Gowda, Jyothi *et al.*, 2003). Plate-like colorless single crystals of (I) were obtained from an ethanolic solution held at room temperature.

### Refinement

All H atoms were positioned geometrically and refined using a riding model, including free rotation about the C_aromatic_–C_methyl_ bond, with C–H = 0.93 or 0.96 Å and N–H = 0.86 Å. The *U*_iso_(H) values were set at 1.2*U*_eq_(C aromatic, N) and 1.5*U*_eq_(C methyl).

### Crystal data

**Table:**

C_15_H_14_ClNO	*F*(000) = 544
*M**_r_* = 259.72	*D*_x_ = 1.335 Mg m^−^^3^
Monoclinic, *P*2_1_/*c*	Mo *K*α radiation, λ = 0.71073 Å
Hall symbol: -P 2ybc	Cell parameters from 11536 reflections
*a* = 13.0108 (5) Å	θ = 2.0–29.4°
*b* = 4.9970 (1) Å	µ = 0.28 mm^−^^1^
*c* = 22.6241 (9) Å	*T* = 295 K
β = 118.553 (4)°	Plate, colorless
*V* = 1292.01 (9) Å^3^	0.54 × 0.43 × 0.05 mm
*Z* = 4	

### Data collection

**Table:**

Oxford Diffraction Gemini R CCD diffractometer	2293 independent reflections
graphite	1959 reflections with *I* > 2σ(*I*)
Detector resolution: 10.434 pixels mm^-1^	*R*_int_ = 0.027
ω scans	θ_max_ = 25.1°, θ_min_ = 2.1°
Absorption correction: analytical (*CrysAlis PRO*; Oxford Diffraction, 2009)	*h* = −15→15
*T*_min_ = 0.861, *T*_max_ = 0.985	*k* = −5→5
20701 measured reflections	*l* = −26→26

### Refinement

**Table:**

Refinement on *F*^2^	Primary atom site location: structure-invariant direct methods
Least-squares matrix: full	Secondary atom site location: difference Fourier map
*R*[*F*^2^ > 2σ(*F*^2^)] = 0.039	Hydrogen site location: inferred from neighbouring sites
*wR*(*F*^2^) = 0.102	H-atom parameters constrained
*S* = 1.06	*w* = 1/[σ^2^(*F*_o_^2^) + (0.0467*P*)^2^ + 0.5282*P*] where *P* = (*F*_o_^2^ + 2*F*_c_^2^)/3
2293 reflections	(Δ/σ)_max_ < 0.001
166 parameters	Δρ_max_ = 0.20 e Å^−^^3^
0 restraints	Δρ_min_ = −0.20 e Å^−^^3^

### Special details

**Table:**

Geometry. All e.s.d.'s (except the e.s.d. in the dihedral angle between two l.s. planes) are estimated using the full covariance matrix. The cell e.s.d.'s are taken into account individually in the estimation of e.s.d.'s in distances, angles and torsion angles; correlations between e.s.d.'s in cell parameters are only used when they are defined by crystal symmetry. An approximate (isotropic) treatment of cell e.s.d.'s is used for estimating e.s.d.'s involving l.s. planes.
Refinement. Refinement of *F*^2^ against ALL reflections. The weighted *R*-factor *wR* and goodness of fit *S* are based on *F*^2^, conventional *R*-factors *R* are based on *F*, with *F* set to zero for negative *F*^2^. The threshold expression of *F*^2^ > σ(*F*^2^) is used only for calculating *R*-factors(gt) *etc*. and is not relevant to the choice of reflections for refinement. *R*-factors based on *F*^2^ are statistically about twice as large as those based on *F*, and *R*- factors based on ALL data will be even larger.

### Fractional atomic coordinates and isotropic or equivalent isotropic displacement parameters (Å^2^)

**Table:**

	*x*	*y*	*z*	*U*_iso_*/*U*_eq_	
C1	0.44821 (16)	0.5493 (3)	0.33073 (9)	0.0402 (4)	
C2	0.32436 (15)	0.4698 (3)	0.28284 (9)	0.0377 (4)	
C3	0.22925 (17)	0.5896 (4)	0.28448 (9)	0.0422 (4)	
C4	0.11586 (17)	0.5209 (4)	0.23906 (10)	0.0525 (5)	
H4	0.0534	0.6029	0.2411	0.063*	
C5	0.09558 (18)	0.3309 (4)	0.19085 (11)	0.0552 (5)	
H5	0.0192	0.2849	0.16	0.066*	
C6	0.18775 (18)	0.2085 (4)	0.18810 (10)	0.0525 (5)	
H6	0.1737	0.0797	0.1554	0.063*	
C7	0.30116 (17)	0.2768 (4)	0.23385 (9)	0.0449 (4)	
H7	0.3631	0.1922	0.2318	0.054*	
C8	0.63848 (15)	0.3676 (3)	0.41456 (9)	0.0392 (4)	
C9	0.68395 (16)	0.5350 (3)	0.47093 (9)	0.0411 (4)	
C10	0.80539 (17)	0.5320 (4)	0.51345 (9)	0.0463 (5)	
C11	0.87516 (17)	0.3630 (4)	0.49984 (10)	0.0523 (5)	
H11	0.9555	0.3622	0.5285	0.063*	
C12	0.82881 (18)	0.1955 (4)	0.44484 (11)	0.0538 (5)	
H12	0.8772	0.081	0.4368	0.065*	
C13	0.71009 (17)	0.1991 (4)	0.40184 (10)	0.0475 (5)	
H13	0.678	0.0882	0.3642	0.057*	
C14	0.6071 (2)	0.7104 (4)	0.48782 (11)	0.0550 (5)	
H14A	0.6218	0.895	0.4827	0.083*	
H14B	0.6242	0.6781	0.5335	0.083*	
H14C	0.5264	0.6697	0.458	0.083*	
C15	0.8603 (2)	0.7101 (5)	0.57434 (11)	0.0662 (6)	
H15A	0.8367	0.8919	0.5611	0.099*	
H15B	0.9439	0.6972	0.5947	0.099*	
H15C	0.8353	0.6549	0.6061	0.099*	
N1	0.51508 (13)	0.3484 (3)	0.36925 (8)	0.0424 (4)	
H1N	0.4817	0.1971	0.3666	0.051*	
O1	0.48384 (12)	0.7779 (3)	0.33335 (7)	0.0546 (4)	
Cl1	0.25124 (5)	0.82447 (11)	0.34617 (3)	0.0650 (2)	

### Atomic displacement parameters (Å^2^)

**Table:**

	*U*^11^	*U*^22^	*U*^33^	*U*^12^	*U*^13^	*U*^23^
C1	0.0486 (11)	0.0274 (9)	0.0474 (10)	−0.0033 (8)	0.0252 (9)	−0.0017 (8)
C2	0.0446 (10)	0.0279 (9)	0.0409 (9)	−0.0020 (7)	0.0206 (8)	0.0043 (7)
C3	0.0518 (11)	0.0332 (9)	0.0453 (10)	0.0006 (8)	0.0262 (9)	0.0016 (8)
C4	0.0452 (12)	0.0519 (12)	0.0599 (12)	0.0072 (9)	0.0249 (10)	0.0069 (10)
C5	0.0457 (11)	0.0538 (13)	0.0517 (12)	−0.0018 (9)	0.0117 (9)	0.0014 (10)
C6	0.0592 (13)	0.0467 (11)	0.0424 (10)	−0.0030 (10)	0.0167 (10)	−0.0061 (9)
C7	0.0496 (11)	0.0388 (10)	0.0474 (11)	0.0016 (8)	0.0241 (9)	−0.0004 (8)
C8	0.0423 (10)	0.0290 (9)	0.0431 (10)	−0.0061 (7)	0.0179 (8)	0.0023 (7)
C9	0.0521 (11)	0.0304 (9)	0.0419 (10)	−0.0070 (8)	0.0233 (9)	0.0028 (7)
C10	0.0532 (12)	0.0390 (10)	0.0389 (10)	−0.0133 (9)	0.0157 (9)	0.0054 (8)
C11	0.0420 (11)	0.0554 (12)	0.0517 (12)	−0.0055 (9)	0.0161 (9)	0.0097 (10)
C12	0.0505 (12)	0.0530 (12)	0.0619 (13)	0.0040 (10)	0.0301 (11)	0.0035 (10)
C13	0.0523 (12)	0.0400 (10)	0.0489 (11)	−0.0034 (9)	0.0232 (10)	−0.0050 (8)
C14	0.0676 (13)	0.0505 (12)	0.0531 (12)	−0.0021 (10)	0.0339 (11)	−0.0029 (10)
C15	0.0742 (16)	0.0584 (14)	0.0483 (12)	−0.0178 (12)	0.0150 (11)	−0.0045 (10)
N1	0.0448 (9)	0.0248 (7)	0.0513 (9)	−0.0071 (6)	0.0178 (7)	0.0000 (6)
O1	0.0565 (8)	0.0259 (7)	0.0734 (10)	−0.0080 (6)	0.0245 (7)	0.0035 (6)
Cl1	0.0793 (4)	0.0553 (4)	0.0724 (4)	−0.0015 (3)	0.0458 (3)	−0.0180 (3)

### Geometric parameters (Å, °)

**Table:**

C1—O1	1.224 (2)	C9—C10	1.403 (3)
C1—N1	1.341 (2)	C9—C14	1.510 (3)
C1—C2	1.503 (3)	C10—C11	1.378 (3)
C2—C7	1.389 (3)	C10—C15	1.503 (3)
C2—C3	1.391 (3)	C11—C12	1.377 (3)
C3—C4	1.379 (3)	C11—H11	0.93
C3—Cl1	1.7395 (19)	C12—C13	1.377 (3)
C4—C5	1.373 (3)	C12—H12	0.93
C4—H4	0.93	C13—H13	0.93
C5—C6	1.374 (3)	C14—H14A	0.96
C5—H5	0.93	C14—H14B	0.96
C6—C7	1.380 (3)	C14—H14C	0.96
C6—H6	0.93	C15—H15A	0.96
C7—H7	0.93	C15—H15B	0.96
C8—C13	1.384 (3)	C15—H15C	0.96
C8—C9	1.398 (2)	N1—H1N	0.86
C8—N1	1.437 (2)		
			
O1—C1—N1	123.65 (17)	C11—C10—C9	119.96 (18)
O1—C1—C2	122.16 (16)	C11—C10—C15	119.49 (19)
N1—C1—C2	114.18 (14)	C9—C10—C15	120.54 (19)
C7—C2—C3	117.59 (17)	C12—C11—C10	121.59 (19)
C7—C2—C1	120.52 (16)	C12—C11—H11	119.2
C3—C2—C1	121.87 (16)	C10—C11—H11	119.2
C4—C3—C2	121.45 (17)	C11—C12—C13	119.30 (19)
C4—C3—Cl1	118.23 (15)	C11—C12—H12	120.3
C2—C3—Cl1	120.29 (14)	C13—C12—H12	120.3
C5—C4—C3	119.67 (19)	C12—C13—C8	120.01 (18)
C5—C4—H4	120.2	C12—C13—H13	120
C3—C4—H4	120.2	C8—C13—H13	120
C4—C5—C6	120.22 (19)	C9—C14—H14A	109.5
C4—C5—H5	119.9	C9—C14—H14B	109.5
C6—C5—H5	119.9	H14A—C14—H14B	109.5
C5—C6—C7	119.95 (19)	C9—C14—H14C	109.5
C5—C6—H6	120	H14A—C14—H14C	109.5
C7—C6—H6	120	H14B—C14—H14C	109.5
C6—C7—C2	121.11 (18)	C10—C15—H15A	109.5
C6—C7—H7	119.4	C10—C15—H15B	109.5
C2—C7—H7	119.4	H15A—C15—H15B	109.5
C13—C8—C9	121.34 (17)	C10—C15—H15C	109.5
C13—C8—N1	116.40 (16)	H15A—C15—H15C	109.5
C9—C8—N1	122.15 (16)	H15B—C15—H15C	109.5
C8—C9—C10	117.77 (17)	C1—N1—C8	124.77 (14)
C8—C9—C14	122.37 (17)	C1—N1—H1N	117.6
C10—C9—C14	119.85 (17)	C8—N1—H1N	117.6
			
O1—C1—C2—C7	118.6 (2)	C13—C8—C9—C14	−176.88 (17)
N1—C1—C2—C7	−60.8 (2)	N1—C8—C9—C14	−0.9 (3)
O1—C1—C2—C3	−59.7 (3)	C8—C9—C10—C11	−1.6 (2)
N1—C1—C2—C3	120.86 (19)	C14—C9—C10—C11	177.02 (17)
C7—C2—C3—C4	−0.3 (3)	C8—C9—C10—C15	179.05 (17)
C1—C2—C3—C4	178.06 (17)	C14—C9—C10—C15	−2.4 (3)
C7—C2—C3—Cl1	177.75 (13)	C9—C10—C11—C12	0.3 (3)
C1—C2—C3—Cl1	−3.9 (2)	C15—C10—C11—C12	179.72 (19)
C2—C3—C4—C5	−0.2 (3)	C10—C11—C12—C13	0.9 (3)
Cl1—C3—C4—C5	−178.28 (16)	C11—C12—C13—C8	−0.8 (3)
C3—C4—C5—C6	0.4 (3)	C9—C8—C13—C12	−0.5 (3)
C4—C5—C6—C7	−0.1 (3)	N1—C8—C13—C12	−176.75 (17)
C5—C6—C7—C2	−0.4 (3)	O1—C1—N1—C8	−5.1 (3)
C3—C2—C7—C6	0.6 (3)	C2—C1—N1—C8	174.31 (16)
C1—C2—C7—C6	−177.78 (17)	C13—C8—N1—C1	−119.2 (2)
C13—C8—C9—C10	1.7 (3)	C9—C8—N1—C1	64.6 (2)
N1—C8—C9—C10	177.69 (15)		

### Hydrogen-bond geometry (Å, °)

**Table:**

*D*—H···*A*	*D*—H	H···*A*	*D*···*A*	*D*—H···*A*
N1—H1N···O1^i^	0.86	2.23	2.9388 (19)	140

Symmetry codes: (i) *x*, *y*−1, *z*.
